# Preliminary Characterization of Proximal Versus Distal Esophageal Function in Healthy, Asymptomatic Adults

**DOI:** 10.1111/nmo.70216

**Published:** 2025-12-04

**Authors:** Erin L. Reedy, Bonnie Martin‐Harris, Jacob Schauer, John E. Pandolfino

**Affiliations:** ^1^ Department of Otolaryngology – Head & Neck Surgery University of Rochester Rochester New York USA; ^2^ Department of Speech Pathology University of Rochester Medical Center Rochester New York USA; ^3^ Department of Communication Sciences and Disorders, Arnold School of Public Health University of South Carolina Columbia South Carolina USA; ^4^ Roxelyn and Richard Pepper Department of Communication Sciences and Disorders, School of Communication Northwestern University Evanston Illinois USA; ^5^ Edward J. Hines, Jr. Veteran's Affairs Medical Center Hines Illinois USA; ^6^ Otolaryngology – Head & Neck Surgery, Radiation Oncology, Feinberg School of Medicine Northwestern University Chicago Illinois USA; ^7^ Biostatistics – Department of Preventative Medicine, Feinberg School of Medicine Northwestern University Chicago Illinois USA; ^8^ Division of Gastroenterology & Hepatology Northwestern University Feinberg School of Medicine Chicago Illinois USA

**Keywords:** deglutition, esophagus, manometry

## Abstract

**Background:**

The reference standard for the assessment of esophageal motility and sphincter function is high‐resolution esophageal manometry (HRM). Diagnostic values for HRM are determined by the Chicago Classification (CC v4.0), which is based almost entirely on distal esophageal function without measures to address the proximal esophageal segment. Therefore, we sought to determine normal HRM values for proximal esophageal function when obtained in the standard HRM positions (supine and upright).

**Methods:**

Healthy, asymptomatic adults (≥ 18 years) were recruited. All participants completed a standard protocol. CC v4.0 measurements, along with a proximal contractile integral (PCI) (millimeters mercury‐seconds‐centimeters[mmHg‐s‐cm]), temporal measures of proximal and distal contractility (seconds), and lengths of proximal and distal esophagus (centimeters), were performed. Summary statistics, tests of normality, and paired two‐sided t‐tests were performed.

**Results:**

HRM data from 30 participants were included. Mean supine PCI was 423.9 mmHg‐s‐cm with a mean contraction time of 3.2 s and a mean length of 5.5 cm. The mean upright PCI was 183.9 mmHg‐s‐cm with a mean contraction time of 2.2 s, and a mean length of 4.5 cm. All proximal values were significantly different comparing the two positions (PCI *p* < 0.0001; time *p* < 0.0001; length *p* < 0.0001). All distal measurements fell within the ranges of normal, and all measures for contractile integral, contraction time, and contraction length were statistically significantly different (*p* < 0.0001 for all) comparing proximal versus distal measurements.

**Conclusions:**

These preliminary data represent our first attempt to quantify normal proximal esophageal function using HRM measurements of contractile vigor, contraction length, and time.

## Summary

- Swallowing pressures in the upper 1/3 (proximal) and lower 2/3rd (distal) esophagus are impacted in seated compared to laying down.
- This article is the first attempt to measure these different pressures using a catheter inserted through the nose and into the esophagus and stomach using a technology called High‐Resolution Esophageal Manometry.

## Graphical abstract

The preliminary data represent the first attempt to quantify normal proximal esophageal function using HRM pressure and temporal contractility measurements in two positions. All proximal values (PCI, contraction time, and contraction length) were significantly different when comparing the two positions (PCI *p* < 0.0001; time *p* < 0.0001; length *p* < 0.0001).

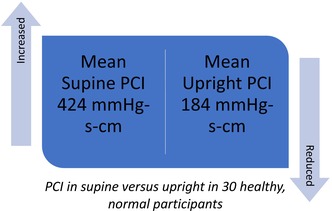

## 1 Introduction

HRM is the reference or “gold” standard for the diagnosis of esophageal motility and sphincter function. Disorders are determined by the Chicago Classification, now on its fourth version (CC v4.0) [1] however, the classification is based almost entirely on distal esophageal function. Therefore, disorders of the proximal esophagus or the “handoff” between the striated and smooth muscle through the transition zone are not currently part of the CC v4.0. Excluding the proximal esophagus in the diagnosis of esophageal swallowing disorders is contrary to the evidence that swallowing is a series of synchronous, overlapping events occurring across a functional continuum (mouth to stomach) [2, 3, 4, 5, 6, 7, 8, 9].

The esophagus is a muscular tube that extends from the pharynx to the stomach. The upper third of the esophagus, or proximal esophagus, consists of striated muscle. The lower two‐thirds of the esophagus, or distal esophagus, is comprised of smooth muscle. Prior to the advent of high‐resolution manometry (HRM), water perfusion manometry systems were used, dating back to the 1970s. Assessment of esophageal function was performed using a “pull‐back” technique to measure pressures of the esophageal body. The proximal esophagus, consisting of striated muscle, was routinely included in the original technique. However, as technologies advanced, it was conceptualized that high‐resolution manometry assessments and classifications would be separated into esophageal disorders and disorders of oropharyngeal and proximal esophageal function (with respective international working groups). When combined, the proximal and distal esophageal measures help to characterize esophageal sphincter (upper esophageal sphincter [UES] and lower esophageal sphincter [LES]) and esophageal body function.

Preliminary data on normal proximal esophageal function in healthy adults exist; however, study protocols and reported measures are variable [10, 11, 12, 13, 14, 15, 16]. Therefore, we aimed to determine a preliminary normal range of values for proximal esophageal function when obtained in the standard HRM positions (supine and upright) and protocol per the current Chicago Classification [1], specifications.

## 2 Methods

### 2.1 Study Design

Study data were collected prospectively as part of an institutional program grant and analyzed retrospectively. The study was conducted at Northwestern University and was approved by the IRB.

### 2.2 Inclusion and Exclusion Criteria

Healthy, asymptomatic adults (≥ 18 years) without a history of gastrointestinal disorders were prospectively recruited as part of a healthy normal database. The database is maintained by the senior author's laboratory in the Gastroenterology and Hepatology department at Northwestern University and Northwestern Memorial Hospital. Any participant with manometric features consistent with disorders of peristalsis or esophagogastric junction outflow obstruction per CC v4.0 was excluded.

### 2.3 High Resolution Manometry Studies

All participants completed 20 swallows of 5 mL liquid (10 supine, 10 upright) delivered via syringe. Standard HRM measurements under the Chicago Classification v4.0 CC v4.0 [1], along with a proximal contractile integral (PCI) (in millimeters mercury‐seconds‐centimeters [mmHg‐s‐cm]), temporal measures of proximal and distal contractility (in seconds), and length of proximal and distal esophagus (in cm) were recorded. Thermal compensation, which aims to correct pressure and temperature differences when the manometry catheter is intubated, was performed prior to analysis. All reported HRM measurements were made with an isobaric contour of 20 mmHg. Studies were analyzed offline using ManoView software (Medtronic, Minneapolis, MN), and all HRM studies were analyzed using the CC v4.0. HRM studies were analyzed by a reliable rater (ELR), and all measurements were made to a single decimal point.

Rules for measuring were decided among co‐authors and operationally defined to ensure consistent analysis of the proximal and distal esophagus. The transition zone was identified at an isocontour of 20 mmHg. We referenced earlier work from this lab that identified the average point of the transition zone at approximately 8 cm during the 4.5 s deglutitive window [12]. Conversely, for participants without a clear landmark, we used the 4.5 s deglutitive window and identified the point at which there was a visually identifiable demarcation (indentation) between 4 and 6 cm to indicate the point of transition and/or to indicate the end of measurable proximal muscle contraction.

**FIGURE 1:**
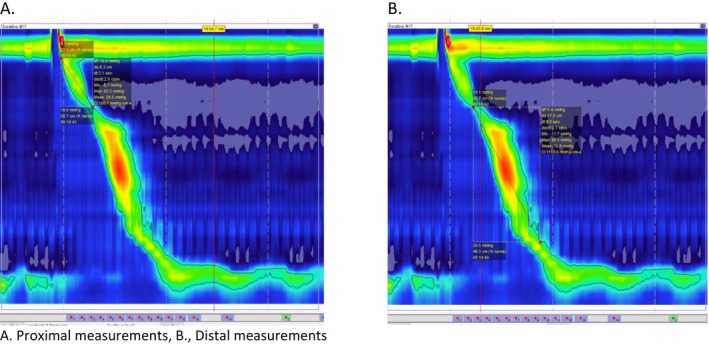
Examples of proximal and distal measurements. (A) Proximal measurements, (B) Distal measurements.

Using the “Smart Tool” feature on the Medtronic software, a box was drawn below the upper esophageal sphincter to the top of the transition zone. Because the transition zone consists of both striated and smooth muscle, we wanted to capture only the striated segment. This single measurement allowed us to capture PCI, proximal segment contraction time, and proximal segment length. Similarly, the distal portion of the esophagus was measured from the top of the transition zone (the point at which the proximal segment ended) to the top of the lower esophageal sphincter/gastroesophageal junction. See (Figure 1).

### 2.4 Statistical Analysis

Descriptive statistics were calculated, and the Shapiro–Wilk W test was used to determine normality of the data. Paired, two‐sided t‐tests were used to compare values in the two study positions (supine and upright). Paired, two‐sample, two‐sided t‐tests were used when comparing compatible proximal versus distal measurements (e.g., proximal contractile integral and distal contractile integral). We report raw *p* values but determined statistical significance using the Sidak adjustment for multiple comparisons, controlling the error rate at 5%. Pearson correlation coefficients (ρ) were used to determine the pairwise association between proximal and distal esophageal measures in the supine versus upright positions.

Findings reflect the overall statistical significance for all statistical analyses. All variables were determined a priori. Analyses were performed using SAS (v9.4, SAS Institute Inc., Cary, NC).

## 3 Results

### 3.1 Participants

Thirty healthy, asymptomatic adults (≥ 18 years) without a history of gastrointestinal disorders were prospectively recruited as part of a healthy normal database. The average age of participants was 28 years (range 19–48). One‐third (33%) were female. Race and ethnicity data were not available for this dataset.

### 3.2 Proximal Esophageal Measurements

Mean supine PCI was 423.9 mmHg‐s‐cm (range 11.8–1706 mmHg‐s‐cm) with a mean contraction time of 3.2 s (range 1.2–6.2 s) and a mean length of 5.5 cm (range 1.5–8.2 cm). The mean upright PCI was 183.9 mmHg‐s‐cm (range 0.4–1589.6 mmHg‐s‐cm) with a mean contraction time of 2.2 s (range 0.5–6.2 s) and a mean length of 4.5 cm (range 0.4–8.1 cm). All proximal values were significantly different when comparing the two positions (*p* < 0.0001 for PCI, time, and length, respectively). See (Table 1).

**TABLE 1:** Esophageal HRM measurements in supine vs. upright positions.

HRM measurements (mean, ±SD, 95% CI)	Supine	Upright	*p*
Proximal measures			
Mean PCI (mmHg‐s‐cm)	423.9 ± 304.1 (389.3, 458.4)	183.9 ± 207.6 (160.1, 207.7)	*p* < 0001*
Mean proximal contraction time (s)	3.2 ± 0.9 (3.1, 3.3)	2.2 ± 0.8 (2.1, 2.3)	*p* < 0001*
Mean proximal length (cm)	5.4 ± 1.2 (4.9, 5.9)	4.3 ± 1.5 (3.7, 5.0)	*p* < 0001*
Distal measures			
Mean DCI (mmHg‐s‐cm)	1501.1 ± 1091.7 (1375.6, 1626.7)	707.7 ± 608.3 (632.8, 782.5)	*p* < 0001*
Mean distal contraction time (s)	6.6 ± 2 (5.8, 7.5)	4.7 ± 2.4 (3.7, 5.8)	*p* < 0001*
Mean distal length (cm)	14.8 ± 2.3 (14.5, 15.1)	12.5 ± 4.1 (12, 13)	*p* < 0001*

*Note:* Paired t‐tests.

* *p* < 05 indicates statistical significance.

Measures of proximal vigor (PCI) were negatively correlated (*ρ* = −0.42) when comparing supine vs. upright contraction. Negative linear correlations were also seen when comparing supine vs. upright proximal contraction time (*ρ* = −0.53) and length (*ρ* = −0.32). Higher values were seen in the supine position, with the PCI 2.3 times greater in the supine versus the upright position.

### 3.3 Distal Esophageal Measurements

Mean supine DCI was 1501.1 mmHg‐s‐cm (range 0–5976.5 mmHg‐s‐cm) with a mean contraction time of 6.6 s (range 0–12.7 s) and a mean length of 14.9 cm (range 0–19.7 cm). The mean upright DCI was 707.7 mmHg‐cm‐s (range 10.4–3081.6 mmHg‐s‐cm) with a mean contraction time of 4.5 s (range 1–11 s), and a mean length of 12.7 cm (range 0–20 cm). All proximal values were significantly different comparing the two positions after multiple comparison adjustment (*p* < 0.0001 for all measures). See (Table 1).

Negative correlations were identified in supine versus upright measures of distal esophageal contractile vigor (DCI) (*ρ* = −0.4), contraction time (*ρ* = −0.53), and length (*ρ* = −0.31). Higher values for DCI were seen in the supine position, with the DCI 2.12 times greater in supine than upright.

### 3.4 Proximal Versus Distal Esophageal Measurements

Both supine and upright measures of contractility (PCI, DCI) were statistically significantly different in the supine (*p* < 0.0001) and upright (*p* < 0.0001) positions, even after multiple comparison adjustment. The other measures of contraction length and contraction time also demonstrated statistically significantly different values. Measures of contraction length (cm) and contraction time (s) both yielded *p* < 0.0001, respectively. The DCI was greater than the PCI in both positions (3.85 times greater in supine, 3.54 times greater in upright). See (Table 2).

**TABLE 2:** Proximal vs. distal HRM measurements.

	Mean PCI (mmHg‐s‐cm)	Mean DCI (mmHg‐s‐cm)	*p*
Supine	423.9	1501.1	*p* < 0.0001*
Upright	183.9	707.7	*p* < 0.0001*

*Note:* Paired t‐tests.

* *p* < 05 indicate statistical significance.

## 4 Discussion

The timing and vigor of proximal esophageal contractions are statistically significantly different when comparing supine versus upright swallows in this healthy cohort. Unsurprisingly, the impact of the proximal esophagus is also significantly different in both positions compared to that of the distal esophagus. This finding confirms the impact of gravity on proximal esophageal function, which has been well established in the distal esophageal musculature [12, 17, 18, 19, 20, 21, 22, 23, 24, 25, 26]. Unlike previous studies examining proximal esophageal measurements [10, 11, 12, 13, 14, 15, 16], our investigation used the protocol and classification system outlined in the Chicago Classification v4.0 [1], the field reference standard, and derived preliminary ranges of normal proximal function in both the supine and upright positions. Though several other studies have reported on proximal esophageal measurements in healthy adults, the study protocols, HRM systems, and reported measures are variable and, therefore, not easily combined for clinical translation. To our knowledge, this is the only study done using the protocol as outlined in the Chicago Classification v4.0 [1].

Normal HRM values for PCI vary greatly in the literature, ranging from 295 mmHg‐s‐cm [11] to 779.2 mmHg‐s‐cm [12]. Our findings report both supine and upright values, with mean values falling within these ranges for our cohort in the supine position (423.9 mmHg‐s‐cm), but outside of these ranges for the upright (183.9 mmHg‐s‐cm). All measures of proximal esophageal and distal esophageal function demonstrated a negative correlation, where the values decrease in the supine position and increase in the upright position. However, the strength of these associations falls within the moderate to weak ranges, which may reflect the limitations of the sample size and certain homogeneity of the participant demographics, as the cohort was relatively young and predominantly male.

It should be noted that each of the prior studies that report proximal measures all used different protocols, with boluses ranging from 1 to 20 mL liquid swallows and two of the studies used additional solid bolus trials [11, 14]. None of the previous studies completed all trials in both supine and upright, representing a gap in the literature which this line of research aims to close.

### 4.1 Influence of Positioning

Positioning had a significant influence on the strength (mmHg‐s‐cm), time of (s), and length (cm) of contractions, with mean PCI values 2.3 times greater in the supine versus the upright position. Similarly, the mean DCI was 2.12 times greater in supine than upright. Comparing proximal to distal contractility, the DCI was approximately 3.85 times greater than the PCI in supine, and 3.54 times greater than the PCI in upright. It appears that the contractile integral increases proportionally within the proximal segment and the distal segment in supine versus upright positioning. A similar proportional increase, albeit greater, occurs between the PCI and the DCI in supine versus upright, where the DCI was 3.54 times greater than the PCI in supine and 3.85 times greater in upright. Differences in supine versus upright have been identified in other measures of HRM. In fact, certain HRM measures may be outside the range of normal for supine swallows but within the normal range for upright swallows, demonstrating a functional “correction” versus disruption of esophageal function depending on positioning [17, 18, 19, 20, 21, 22, 23, 24, 25, 26, 27]. Positional differences in pressure have been identified in the high‐resolution pharyngeal manometry (HRPM) literature, where Rosen and colleagues [28], identified significant alterations to velopharyngeal pressures in this study that used HRPM with participants' positions measured in upright and between 45° and 180°. The study also investigated changes to UES pressures (pre‐ and post‐opening maximum, nadir, and minimum pressures), with only the minimum pressures demonstrating statistically significant differences (greatest at 45° and 90° compared to upright and 180°).

Gravity is a significant facilitator of esophageal function and bolus clearance [17, 18, 23, 27], which is why all reference standard esophageal testing includes at least a portion of the test supine. The elimination of gravity, while necessary to include in esophageal testing, does not reflect the conditions for typical human swallowing. Therefore, including upright swallows in esophageal assessment is essential. As our knowledge of the influence on positioning the esophageal body grows, this may also lead to future revisions to the classification for esophageal disorders. Including proximal esophageal function may help to further characterize and/or differentially diagnose esophageal motility disorders.

### 4.2 Limitations

There are several limitations to this study. First, the sample size is small and consists of relatively younger adults. Second, owing to the timeframe when the studies were completed, impedance was not available for this cohort. Without multichannel intraluminal impedance (MII), we were unable to characterize esophageal clearance for liquid swallows in our healthy cohort, which may provide valuable insight into the impact of proximal esophageal muscle function in bolus transport through the esophageal body. Finally, there is evidence that traditional HRM may underestimate the contractility of the proximal esophagus owing to the anterior‐superior movement of hyolaryngeal structures and shortening of the longitudinal pharyngeal constrictors during a swallow that displaces the UES and proximal esophagus [29].

### 4.3 Future Directions

We aim to continue this work in a larger cohort of healthy, asymptomatic adults to further help characterize normal proximal and esophageal function across adulthood. To best understand disease, we must be able to fully characterize the definitions and ranges of what is expected in healthy, normal individuals. Therefore, impedance (HRM‐MII) will be utilized in future studies to help further characterize normal esophageal function in adults. Additional considerations for future studies include simultaneous manometry and fluoroscopy (mano‐fluoro) as well as the possibility for planimetry (e.g., endoFLIP) to assess the UES and proximal esophagus.

Our future work is directed toward closing the gap in understanding between the relationships of proximal and distal esophageal function in health. This line of research will ultimately focus on uncovering the contribution of proximal esophageal measures to the overall quantification of esophageal impairment in esophageal diseases and disorders.

## 5 Conclusion

These preliminary data represent the first attempt to quantify normal proximal esophageal function using HRM pressure and temporal contractility measurements in a small cohort. This pilot data and future work are directed toward closing the gap in understanding between the relationships of proximal and distal esophageal function in health and disease.

## Author Contributions

Study design, data acquisition, and drafting of the original manuscript were performed by the first author, E.L.R. Both E.L.R. and J.S. completed data analysis and contributed to the interpretation of this work. B.M.‐H. and senior author, J.E.P., contributed substantially to the study's conceptualization and editing of earlier versions of the manuscript. All authors read and approved the final manuscript.

## Funding

This work was funded in part by VA RR&D, 1I01RX002352‐01A1, “Clinical Impact of Respiratory Swallow Training on Refractory Dysphagia in OP HNC”, 2018–2024 (PI: Martin‐Harris) and NIH/NIDDK, 5P01DK117824–05 “Disordered Tissue Biomechanics as a Driver of Esophageal Disease” (PI: Pandolfino). Erin Reedy and Jacob Schauer have no competing interests to disclose.

## Disclosure

Dr. Martin‐Harris holds a U.S. provisional patent (Feb 16, 2018: US 62/710,324).

Inventors: Shuai Xu, Kun Lee, Angela Roberts, Bonnie Martin‐Harris, and John Rogers. Dr. Pandolfino sits on the Advisory Board and receives speaking honoraria for Phathom Pharmaceuticals, speaking honoraria, and consulting fees from Medtronic. Dr. Pandolfino holds U.S. patents (January 2021: US‐20180008156‐A1, Inventors: Pandolfino, Lin, Kahrilas, O'Dea, and McHugh; January 2018: US‐10898091‐B2, Inventors: Pandolfino, Lin, Kahrilas, O'Dea, and McHugh).

## Conflicts of Interest

The authors declare no conflicts of interest.

## Data Availability

The data that support the findings of this study are available from the corresponding author upon reasonable request.
